# Neuropeptide Y receptors: how to get subtype selectivity

**DOI:** 10.3389/fendo.2013.00005

**Published:** 2013-02-04

**Authors:** Xavier Pedragosa-Badia, Jan Stichel, Annette G. Beck-Sickinger

**Affiliations:** Institute of Biochemistry, Faculty of Biosciences, Pharmacy and Psychology, Universität LeipzigLeipzig, Germany

**Keywords:** GPCR, NPY, YR, subtype selectivity, ligand side, receptor side

## Abstract

The neuropeptide Y (NPY) system is a multireceptor/multiligand system consisting of four receptors in humans (hY_1_, hY_2_, hY_4_, hY_5_) and three agonists (NPY, PYY, PP) that activate these receptors with different potency. The relevance of this system in diseases like obesity or cancer, and the different role that each receptor plays influencing different biological processes makes this system suitable for the design of subtype selectivity studies. In this review we focus on the latest findings within the NPY system, we summarize recent mutagenesis studies, structure activity relationship studies, receptor chimera, and selective ligands focusing also on the binding mode of the native agonists.

## INTRODUCTION TO THE NEUROPEPTIDE Y FAMILY

The neuropeptide Y (NPY) family is a multireceptor/multiligand system consisting of four receptors in humans and three polypeptides that bind and activate them with different affinity and potency. The NPY receptors belong to the class A or rhodopsin-like G-protein coupled receptors (GPCR). Five receptors have been cloned from mammals so far, Y_1_, Y_2_, Y_4_, Y_5,_ and y_6_ but only four of the members are functional in humans (hY_1_, hY_2_, hY_4_, hY_5_; **Table 1**). The y6 receptor however is active in rabbit and mouse (Starback et al., 2000). The existence of an additional receptor subtype (Y_3_) was suggested by pharmacological studies of several human, rat, and rabbit tissues including the human adrenal medulla. This receptor subtype is characterized by a much lower affinity for PYY, compared to NPY (Gehlert, 1998; Lee and Miller, 1998). However, since all attempts to clone this receptor subtype were unsuccessful so far, the existence of Y_3_ is not very likely.

**Table 1:** Amino acid sequence of the NPY ligands.

Peptides	Amino acid sequence																																			
	1				5					10					15					20					25					30					35	
pNPY	Y	P	S	K	P	D	N	P	G	E	D	A	P	A	E	D	L	A	R	Y	Y	S	A	L	R	H	Y	I	N	L	I	T	R	Q	R	Y
hPYY	Y	P	I	K	P	E	A	P	G	E	D	A	S	P	E	E	L	N	R	Y	Y	A	S	L	R	H	Y	L	N	L	V	T	R	Q	R	Y
hPP	A	P	L	E	P	V	Y	P	G	D	N	A	T	P	E	Q	M	A	Q	Y	A	A	D	L	R	R	Y	I	N	M	L	T	R	P	R	Y

Neuropeptide Y receptors (NPYR) generally couple to G_i_ or G_0_ proteins, which leads to the inhibition of adenylate cyclase and finally to the inhibition of cAMP accumulation (Cabrele and Beck-Sickinger, 2000) and modulation of Ca^2^^+^ and K^+^ channels (Holliday et al., 2004). Besides this, it has been described that Y_2_ and Y_4_ receptors also couple to the G_q_ protein increasing inositol 1,4,5-phosphate (IP_3_) production via the activation of the phospholipase C-β (PLC) in rabbit smooth muscle cells (Misra et al., 2004).

Neuropeptide Y, peptide YY (PYY), and pancreatic polypeptide (PP) are the native ligands of the NPY family. NPY is the most abundant peptide in the mammalian brain and has been suggested to adopt a largely open structure. In surface association with phospholipid micelles a flexible N-terminus and a C-terminal alpha helix were identified (Lerch et al., 2004; Parker et al., 2011). However, PYY and PP are suggested to form the typical hairpin-like structure also called PP-fold, a suggestion for pPYY supported by NMR (Keire et al., 2000a; Neumoin et al., 2007), and for PP by the X-ray structure of the peptide (Blundell et al., 1981). Despite some structural differences between the ligands, these polypeptides have a common length of 36 amino acids (**Table 2**) and an amidated C-terminus. Furthermore, these polypeptides share high sequence identity. Whereas NPY and PYY show the highest percentage of common residues with 70%, NPY and PP share only 50% identity (Blomqvist et al., 1992; Zhang et al., 2011). Seven positions in NPY, PYY, and PP are strongly conserved throughout all species: Pro^5^, Pro^8^, Gly^9^, Ala^12^, Tyr^27^, Arg^33^, and Arg^35^. Apart from these, highly conserved positions are: Pro^2^, Tyr^20^, Thr^32^, and Tyr^36^ (Cabrele and Beck-Sickinger, 2000). Regarding its pharmacological properties, NPY acts as a neurotransmitter whereas PYY and PP act as neuroendocrine hormones.

**Table 2:** NPYR: sequence length and ligand preference.

Receptor	hY_1_	hY_2_	hY_4_	hY_5_
Amino acids number	384	381	375	445–455
Native ligand	NPY	NPY	PP	NPY
	PYY	PYY		PYY

The first identified member of the family PP, was isolated from avian pancreas in 1975 (Kimmel et al., 1975). This polypeptide is secreted in the pancreas by PP cells in the Langerhans islets after food ingestion in proportion to the caloric content (Boguszewski et al., 2010; Suzuki et al., 2010). It is thought to act mainly in brain stem and vagal nerve where it promotes appetite suppression, inhibition of gastric emptying and increases in energy expenditure (Asakawa et al., 2003) in addition to direct responses in the gut.

The second member of the ligand family PYY, was isolated from porcine intestinal extracts in 1980 (Tatemoto and Mutt, 1980) and is expressed by entero-endocrine L cells of the distal gut (Lundberg et al., 1982). PYY_1-36_ is released in proportion to nutrient intake along the gut and cleaved to PYY_3-36_ by the dipeptidyl aminopeptidase VI. The ligand PYY_3-36_, the predominant form released in the circulation, is selective for Y_2_ and produces anorexigenic effects (Pittner et al., 2004). This polypeptide acts on peripheral receptors but also on those located in the CNS (Hankir et al., 2011; Schloegl et al., 2011). The last family member, NPY, was isolated from porcine brain in 1982 (Tatemoto, 1982) and is one of the most broadly distributed peptides of the central and peripheral nervous system. This peptide is well conserved among different species. It stimulates food intake in response to negative energy balance (Stanley et al., 1986). Additional roles of NPY are decreased bone formation (Baldock et al., 2009; Sousa et al., 2012), regulation of mood and anxiety disorders, the modulation of stress responses (Heilig, 2004), and ethanol intake (Thiele et al., 1998).

Neuropeptide Y family peptides mediate their activity in humans via four receptors. Structurally, these receptors contain two Cys residues in the extracellular regions that form a disulfide bond between extracellular loop I and II. This disulfide bond is a common feature of class A GPCRs and has been confirmed by X-ray crystallography for several members including bovine rhodopsin and the human β2 adrenergic receptor (Palczewski et al., 2000; Cherezov et al., 2007).

The evolution of this system shows that vertebrate ancestors probably had three receptor genes. These genes, possibly located in close proximity in the same chromosomal segment, would be the precursors of the receptor subfamilies. The Y_1_ subfamily includes the Y_1_, Y_4_, and y_6_ receptors, the Y_2_ subfamily comprises Y_2_ and Y_7_ (in zebrafish and frogs), and the Y_5_ subfamily consists of only the Y_5_ due to lack of close relatives of this receptor (Larhammar and Salaneck, 2004). Although the Y_1_ and Y_2_ receptor subtypes have a common pharmacological profile, they only share 27% of sequence identity. Y_1_ and y_6_ receptors share the highest sequence identity (51%), whereas Y_1_ and Y_4_ receptors share 44% identity, increasing to 56% identity in transmembrane regions (Larhammar et al., 2001). The Y_4_ receptor conserves 75% of overall identity between human and rat suggesting this protein may be the most rapidly evolving member of the family and the only member that has a selective agonist, the pancreatic polypeptide (Larhammar, 1996; Blomqvist and Herzog, 1997). The Y_5_ receptor displays low sequence identity, around 30%, with all members of the family. Compared with other GPCRs, neuropeptide Y receptors share a high sequence identity with NPFF_1_ and NPFF_2_ receptors, which are members of the RFamide receptor family (Bonini et al., 2000).

The Y_1_ receptor has 384 amino acids and its main agonists are NPY and PYY. It can be also activated by PP with a minor potency (**Table 2**). The receptor is expressed in the hypothalamus, hippocampus, neocortex, and thalamus (Caberlotto et al., 1997), but is also present in adipose tissue (Castan et al., 1993; Hausman et al., 2008), blood vessels (Cabrele and Beck-Sickinger, 2000), colon, kidney, adrenal gland, heart, and placenta (Wharton et al., 1993). It plays a role in the regulation of food intake (Kanatani et al., 2000b), vasoconstriction of blood vessels (Cabrele and Beck-Sickinger, 2000), heart rate, anxiety (Balasubramaniam, 2002), and bone homeostasis (Sousa et al., 2012).

The Y_2_ receptor is predominantly expressed in hippocampal neurons, in the thalamus, hypothalamus, and parts of the peripheral nervous system (Widdowson, 1993; Cabrele and Beck-Sickinger, 2000). It is mainly found in pre-synaptic neurons and exerts its action through the regulation of neurotransmitter release (Wahlestedt et al., 1986; Potter et al., 1989). Typical effects correlated with activation of this receptor include enhanced memory retention, the regulation of the circadian rhythm, angiogenesis (Flood and Morley, 1989; Golombek et al., 1996; Gribkoff et al., 1998; Zukowska-Grojec et al., 1998) and bone formation (Baldock et al., 2002). This receptor consists of 381 amino acids and its preferred agonists are NPY and PYY (**Table 2**).

The Y_4_ receptor subtype is the only member of the family with the endogenous agonist PP, while PYY and NPY can still activate this receptor with minor potency (**Table 2**). It consists of 375 amino acids and is mainly expressed in the gastrointestinal tract (Lundell et al., 1995; Ferrier et al., 2002) but also in the brain (Bard et al., 1995), as well as pancreas and prostate (Lundell et al., 1995). It plays a role in the regulation of feeding (Asakawa et al., 1999; Sainsbury et al., 2010), circadian ingestion and energy homeostasis (Edelsbrunner et al., 2009), colonic transit (Moriya et al., 2010), and stimulation of the luteinizing hormone release (Jain et al., 1999).

The Y_5_ receptor subtype is expressed in two different splice variants, composed of 445 and 455 amino acids, respectively (**Table 1**). The N-terminus of the longer isoform is extended by 10 amino acids. However, these differences in the sequence of the receptor isoforms do not result in differences in their pharmacological profile (Rodriguez et al., 2003). Both receptor isoforms bind NPY and PYY with comparable affinities. The affinity for PP is slightly lower, but still in the nanomolar range (Gerald et al., 1996). Y_5_ receptors are mainly expressed in the central nervous system. Tissues with high receptor density include the hippocampus and hypothalamus. The Y_5_ receptor subtype has been shown to be strongly involved in food intake (Gerald et al., 1996). Other possible roles of the Y_5_ receptor are the regulation of the circadian rhythm (Matsumoto et al., 1996b; Gribkoff et al., 1998) and reproduction through inhibition of LH release (Raposinho et al., 2001).

The y6 receptor encodes a 371 amino acid protein that has been cloned from rabbit, mouse, and chicken among others (Bromee et al., 2006). However, the sequence in humans and monkeys contains a frame shift mutation in the third intracellular loop, resulting in a non-functional truncated receptor protein (Matsumoto et al., 1996a; Michel et al., 1998).

Taken together, this multireceptor/multiligand system mediates many relevant physiological and pathological processes. This makes the NPY family truly attractive for the design of subtype selective analogs and receptors. Even if selective ligands are pharmacologically the most attractive approach to tackle subtype selectivity, development of receptor chimeras or receptor mutants will also help to understand how the receptors refined their binding pockets during evolution and, as a result, will show how the ligands tend to have distinct affinities for one or the other receptor subtype.

## DEVELOPING SELECTIVE LIGANDS FOR NPY RECEPTORS

As it has been previously described, the binding affinity of each peptide differs from receptor to receptor and the role that each receptor plays in regulating physiological processes is different. In light of this, the NPY system is a perfect candidate in which to develop selective ligands and selective receptors to modulate these characteristics.

### GENERAL STRATEGIES

The most conventional way of investigating subtype selectivity is the synthesis of selective ligands. Consequently, to obtain subtype selective ligands, the peptides have to be modified in key positions allowing the investigator to modulate the ligand preference for a receptor. Although the peptides of the NPY family share high sequence homology, they do not necessarily have the same binding mode. The truncation of certain fragments can direct the selectivity to a certain receptor subtype providing information about essential fragments of the peptide. Therefore, one of the approaches to investigate important positions on the peptides are N- or C- terminal truncations.

Another approach to investigate subtype selectivity is the alanine-scan or Ala-scan: this means that each residue in the sequence is one by one individually substituted with Ala. When an Ala occurs naturally in a certain position, this residue is then changed to Gly. In this scan, only the functional groups are substituted permitting the investigation of ionic interactions as well as dipole-dipole and hydrophobic interactions. Once all the analogs are synthesized they must be tested at all the receptor subtypes to determine how the substitution of the native amino acid affects the binding or the activation. In case a residue shows a great loss in binding or activation for a certain receptor, further exchanges in this position can be done. For example the exchange of a certain residue of NPY by the residue present in PP can achieve Y_4_ receptor binding with the analog. The use of D-amino acids in a scan can provide information about the side chain orientation and steric information concerning ligand binding too, Pro-scans reveal favorable turn-structures and Phe-scans hydrophobic interactions (Lindner et al., 2008a).

As small peptides can adopt several active conformations and these conformations can be recognized by different receptor subtypes in structure-activity relationships, the knowledge of these binding subtypes is of great interest. Furthermore, to investigate the binding mode and receptor preference of small antagonists or non-peptidic drugs, knowledge of the bioactive conformation is of major importance. Constraining the ligand conformation and testing the peptide on several receptor subtypes, can provide information about its bioactive conformation and receptor selectivity. Several strategies can be used to investigate structure activity relationships constraining the conformation of small peptides (Beck-Sickinger, 1997). First of all, non-proteinogenic amino acids can be incorporated, reducing the number of angle combinations that a natural amino acid could adopt, and thereby decreasing the flexibility of the peptide. One example of a non-proteinogenic amino acid is Aib (aminoisobutyric acid). This residue is one the most commonly used in this kind of study. Secondly, the use of several templates and amino acid linkers to induce a desired conformation might be also a good strategy, although this does not always lead to the desired effect because of other amino acids within the sequence. The use of more flexible linkers such as Ahx (6-aminohexanoic acid) or ω-amino alkanoic acids might be a better method to determine the distance between two segments. Finally, the use of cyclization can significantly constrain the conformation of a ligand. Several cyclization techniques can be applied, the most commonly used are: cyclization by disulfide formation between two Cys residues, cyclization by lactamization of N- and/or C-terminus or by the N- and C-group-containing side chains Lys, Orn, Dab, Asp, and Glu and backbone to side-chain cyclization. Recent studies also use click reactions to cyclize peptides using triazoles to mimic disulfide bridges (Holland-Nell and Meldal, 2011) and peptide stapling to increase the propensity to form α-helices, therefore improving pharmacological properties (Verdine and Walensky, 2007).

### Y_1_ RECEPTOR

N-/C-terminal truncations of NPY confirm the importance of these two segments for Y_1_ receptor binding. N-terminally truncated analogs are not well accepted by the Y_1_ receptor as can be seen in studies using the shortened sequences NPY(3–36), (13–36), and (18–36). These show only micromolar affinities for this receptor and even the truncation of the first amino acid NPY(2–36), results in a loss of affinity (Beck-Sickinger and Jung, 1995). C-terminal truncations show the importance of the amide group in the binding with the receptor (Hoffmann et al., 1996). Centrally truncated analogs containing the spacer Ahx and structurally constrained analogs showed that the N- and C-terminal fragments must have a certain length to bind with a good affinity to the receptor (Kirby et al., 1993b). Furthermore, using an Ala-scan it was found that, Pro^2^, Pro^5^, Arg^19^, and Tyr^20^ are important for ligand affinity. Also the amino acids from positions 27 to 36 were found to be crucial for the peptide, especially position 27. Moreover, positions 33 and 35 showed to be extremely important, as Ala analogs at these positions produced a dramatic loss in binding of>5000-fold over wt (**Figure 3**; Beck-Sickinger et al., 1994; Cabrele and Beck-Sickinger, 2000; Lindner et al., 2008b). The importance of Arg^35^ was further confirmed as this residue was found to form a subtype-specific ionic interaction with Asp^6.59^ of the receptor (Merten et al., 2007). The Tyr on position 36 was also found to be relevant for the ligand binding; this position does however tolerate the exchange to Phe, but not Ala, Bpa, or His. Similar results were obtained using a D-amino acid scan (Kirby et al., 1993a).

**FIGURE 1:**
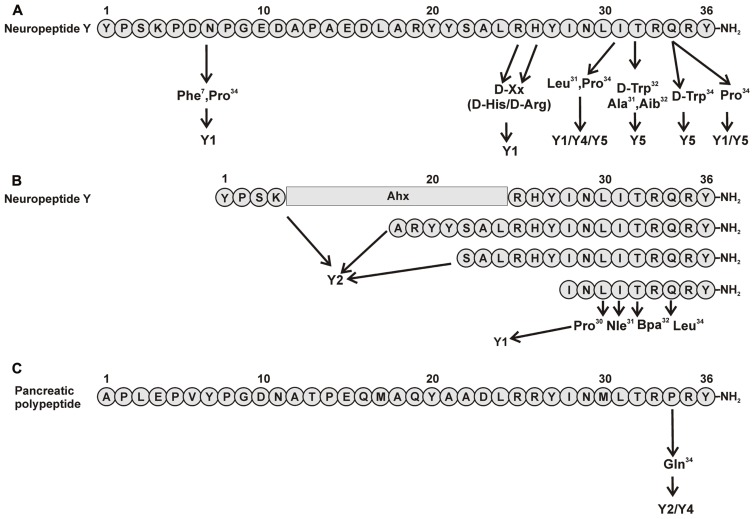
**Important amino acid positions and truncated peptides to introduce selectivity to NPY receptors**. **(A)** Important positions in pNPY (Fuhlendorff et al., 1990; Cabrele et al., 2000, 2002; Parker et al., 2000; Mullins et al., 2001; Soll et al., 2001); **(B)** Truncations of pNPY (Beck et al., 1989; Fournier et al., 1994; Beck-Sickinger and Jung, 1995; Keire et al., 2000b; Zwanziger et al., 2009). **(C)** Important positions in hPP (Schwartz, 2006).

Positions 7, 25, 26, 31, and 34 were revealed to be important for subtype selectivity (**Figure 1A**). Modifications in positions 25 and 26 showed that [D-Arg^25^]NPY and [D-His^26^]NPY bind selectively to the Y_1_ receptor (Mullins et al., 2001). Also the introduction of Pro in position 34, present in pancreatic polypeptide, redirected the affinity of the peptide to Y_1_/Y_5_ receptors. Apart from Gln^34^, an additional exchange in Asn^7^ introducing Phe at this position, a similar residue like the Tyr present on the hPP, yielded [Phe^7^, Pro^34^]pNPY. This is a selective Y_1_ receptor binder and illustrated the importance of an aromatic residue in this position (Soll et al., 2001). Also the combination of Pro^34^ with an exchange in position 31 by Leu contributes to aY_1_/Y_4_/Y_5_receptor selective profile (Fuhlendorff et al., 1990; Cabrele et al., 2000). All this strongly indicates the importance of N- and C-terminal fragments for the Y_1_ receptor subtype.

**FIGURE 2:**
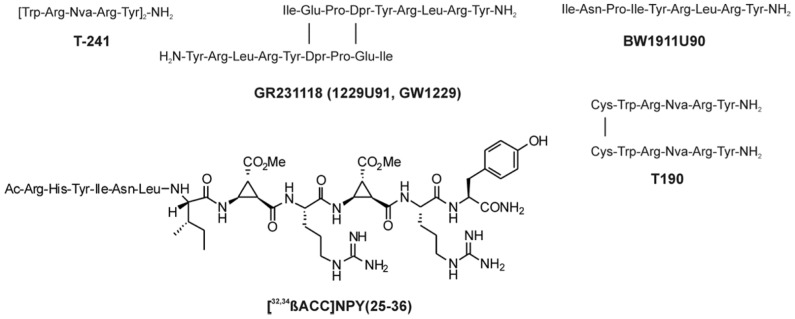
**Peptidic antagonists of the Y_1_ receptor **(Parker et al., 1998; Koglin et al., 2003).

The synthesis of small selective ligands has also been a topic of interest in the past years and many peptides have been synthesized and characterized. Although the first experiments with short- or medium-sized pNPY truncations showed low binding affinity at the hY_1_ receptor, in recent years several short antagonists, mimicking the NPY C-terminus have been synthesized such as, GR231118 (1229U91 or GW1229), T-241, and T-190. Unfortunately, these ligands also have Y_4_ agonistic properties (**Figure 2**; Parker et al., 1998). Taking the short NPY analog NPY (28–36) and the antagonist GR231118, Zwanziger et al. (2009) designed a set of 19 short peptide analogs. Only [Pro^30^, Nle^31^, Bpa^32^, Leu^34^]NPY(28–36) displayed hY_1_ receptor selectivity and was able to activate the receptor (**Figure 1B**). Follow-up investigations were made by Hofmann and colleagues (Neuropeptides, accepted) on position 32. The authors could further stabilize the peptide by replacing Bpa by Bip (biphenylalanine) and could switch the activity from hY_1_ receptor to hY_2_/hY_4_ receptors by introducing an ortho-carbaboranyl moiety. Other small peptide antagonists are BW1911U90 and [^32^^-^^34^βACC]-NPY(25–36)]; **Figure 2**; Koglin et al., 2003), and examples of known non-peptidic antagonists are BIBP3226, BIBO3304, LY357897, J-104870 (**Figure 4A**; Rudolf et al., 1994; Hipskind et al., 1997; Wieland et al., 1998; Sjodin et al., 2006; Antal-Zimanyi et al., 2008).

### Y_2_ RECEPTOR

**FIGURE 3:**
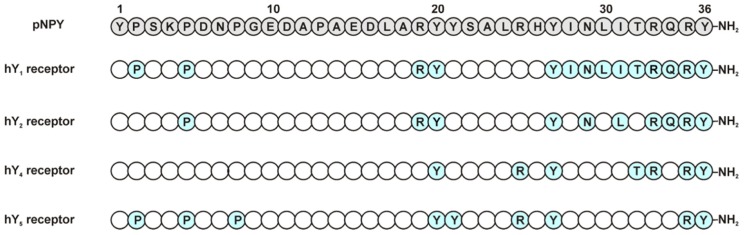
**Relevant amino acids in pNPY Ala-scan at NPY receptors**. Positions in blue represent the amino acids showing a high impact when exchanged to Ala (Beck-Sickinger et al., 1994).

As with the human Y_1_ receptor, the Y_2_ receptor binds NPY and PYY with comparable affinities. Beside these two native high-affinity ligands, a number of Y_2_-selective NPY-derived peptide agonists have been synthesized in the past. Interestingly, in contrast to all other Y receptors, Y_2_ receptors allow large truncations of the peptidic ligands without loss of affinity (Beck-Sickinger and Jung, 1995) and also cyclizations between N- and C-terminally located residues are tolerated (Kirby et al., 1993b). Most commonly used Y_2_ receptor selective NPY-analogs are the N-terminally truncated NPY(3–36) and NPY(13–36). Even larger N-terminal truncations and centrally truncated analogs can bind to the Y_2_ receptor with nanomolar affinity [e.g., NPY(18–36), NPY(22–36), [Ahx^5^^-^^24^]NPY; **Figure 1B**; Beck et al., 1989; Fournier et al., 1994; Beck-Sickinger and Jung, 1995; Keire et al., 2000b]. An Ala-scan of the complete NPY peptide revealed only few positions to be highly important (**Figure 3**).

**FIGURE 4:**
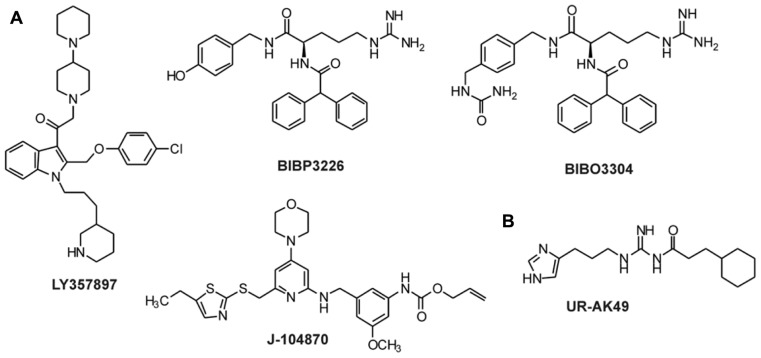
**Non-peptidic antagonists for Y_1_ and Y_4_ receptors**. **(A)** Antagonists for the Y_1_ receptor (Rudolf et al., 1994; Hipskind et al., 1997; Wieland et al., 1998; Balasubramaniam et al., 2001; Sjodin et al., 2006; Antal-Zimanyi et al., 2008); **(B)** Antagonists for the Y_4_ receptor (Ziemek et al., 2007).

The substitution of Pro^5^ to Ala led to a 600-fold loss of affinity. Accordingly, all other important residues except Pro^5^ are located in the C-terminal part of NPY. The individual substitution of Arg^19^, Tyr^20^, Tyr^27^, and Asn^29^ in the NPY peptide showed a 30- to 40-fold lower affinity. A more dramatic effect could be observed for the residues Leu^31^ (1000-fold lower affinity), Arg^33^ (1350-fold), Gln^34^ (150-fold), Arg^35^ (75000-fold), and Tyr^36^ (17500-fold; **Figure 3**; Cabrele and Beck-Sickinger, 2000; Eckard et al., 2001). Interestingly, the introduction of a Pro residue at position 34 is not tolerated at the Y_2_ receptor, which is in contrast to the effect observed on the other Y receptor subtypes (Beck-Sickinger et al., 1994; Keire et al., 2000b; Eckard et al., 2001). Although Tyr^36^ may not be substituted by Ala, the introduction of Hty (homotyrosine) or p-substituted Phe in PYY(3–36) is well tolerated at the Y_2_ receptor, but almost completely abolishes binding of the modified NPY analogs at Y_1_ or Y_4_ receptors (Pedersen et al., 2009). Taken together, these data underline the importance of the C-terminal part of the peptide ligand for high-affinity binding to the Y_2_ receptor, despite the fact that the binding pocket for NPY at the Y_2_ receptor seems to be less narrow than the ones of Y_1_ or Y_4_ receptors.

**FIGURE 5:**
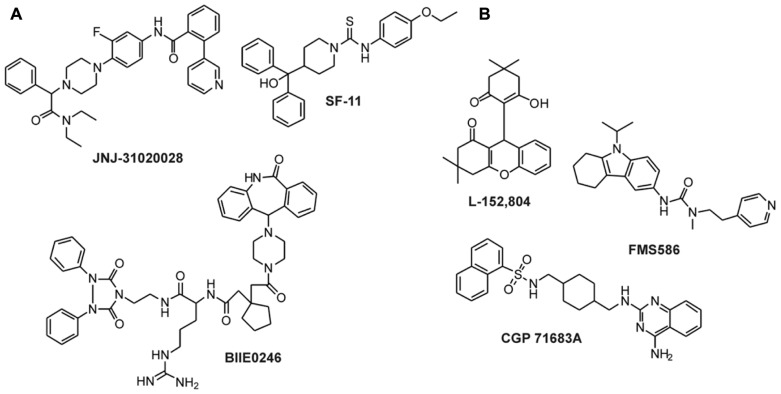
** Non-peptidic antagonists of Y_2_ and Y_5_ receptors**. **(A)** Y_2_ receptor antagonists (Doods et al., 1999; Saldanha et al., 2009; Shoblock et al., 2010; Swanson et al., 2011); **(B)** Y_5_ receptor antagonists (Criscione et al., 1998; Kanatani et al., 2000a; Rueeger et al., 2000; Kakui et al., 2006).

A number of selective high-affinity antagonists at the Y_2_ receptor have been published so far. The most widely used compound in pharmacological studies is BIIE0246 (**Figure 5A**; Doods et al., 1999). In order to identify compounds with improved biostability, bioavailability, and brain permeability, further studies have been conducted. A number of molecules and scaffolds have been reported as highly selective and affine small molecule Y_2_ receptor antagonists (**Figure 5A**) including JNJ-527787 (Bonaventure et al., 2004; Jablonowski et al., 2004), SF-11, SF-21, SF-22, SF-31, SF-41 (Brothers et al., 2010), ML072 to ML075 (Saldanha et al., 2009), JNJ-31020028 (Shoblock et al., 2010; Swanson et al., 2011), a series of substituted 3-chloranilides (Lunniss et al., 2009, 2010), CYM 9484, and CYM 9552 (Mittapalli et al., 2012).

### Y_4_ RECEPTOR

The Ala-scan of the NPY (Eckard et al., 2001) revealed that again Arg^33^ and Arg^35^ are crucial for receptor affinity. Ala substitutions in these positions led to a dramatic loss in binding. Positions Tyr^20^, Tyr^27^, Arg^25^, Thr^32^, and Tyr^36^ are also important residues in the ligand and showed a loss in binding affinity (30- to 60-fold), whereas Pro^5^, Pro^8^, and Tyr^21^ proved to be less relevant, causing only a slight loss in affinity (5- to 10-fold) when changed to Ala (**Figure 3**).

In a follow-up study using hPP (Merten et al., 2007), Arg residues 33 and 35 were confirmed to be essential for receptor activation, showing a dramatic effect when exchanged to Ala in position 33. Position Arg^35^ was found to interact with Asp^6.59^ of the receptor.

Because this receptor subtype has its own selective ligand, peptide research is more focused on improving proteolytic stability and increasing bioavailability of the peptide. However, a number of specific ligands have been published in the past years for this receptor. As previously described, position 34 of NPY peptides is a key residue to introduce Y_4_ receptor selectivity to NPY and PYY, whereas in PP when exchanging Pro^34^ for Gln the peptide acquires Y_2_ agonistic properties without losing Y_4_ receptor activity. Some of the analogs published like [Gln^34^]-hPP, the so called Obinepitide (Schwartz, 2006), which is selective for Y_2_ and Y_4_ receptors, contains this exchange (**Figure 1C**). Other small peptide agonists described also as Y_1_ receptor antagonists are: GR231118 (1229U91 or GW1229), T-241, and T-190 (**Figure 2**; Schober et al., 1998).

To our knowledge, only one ligand with antagonistic properties at the hY_4_ receptor has been published up to now. UR-AK49 (**Figure 4B**) is a weak hY_4_ receptor antagonist but unselective, because it can also bind to hY_1_ and hY_5_ receptors (Ziemek et al., 2007).

### Y_5_ RECEPTOR

The hY_5_ receptor subtype does not tolerate large truncations of NPY. While the deletion of the first amino acid is accepted by the hY_5_ receptor, further N-terminal truncation of NPY results in a decreased affinity of the peptides. Similarly, larger central truncations of NPY are not tolerated by Y_5_ receptors. The only centrally truncated analog of NPY with high Y_5_ receptor affinity is [Ahx^9-17^]pNPY with a ~15-fold decreased affinity compared to pNPY (Cabrele and Beck-Sickinger, 2000).

An Ala scan of the complete peptide revealed the Pro residues 2, 5, and 8 to be important for the affinity of NPY at the Y_5_ (**Figure 3**). Haack et al. (2008) could confirm the importance of the peptide N-terminus for high-affinity binding at the Y_5_ receptor by a pyridone dipeptide scan. In addition to these findings, the individual substitution of Tyr residues 20, 21, 27, and 36 to Ala led to a loss of affinity; Arg^25^ was also shown to be important for the ligand affinity. The highest impact could be observed for Tyr^27^ (~400-fold) and Arg^35^ (1000-fold; Cabrele and Beck-Sickinger, 2000), these findings fit with the fact that these two positions are involved in interactions with the receptor.

A number of selective high-affinity analogs of NPY and PYY have been developed in the past. It has been shown that especially the substitution of position 32 of the peptide ligands is critical for Y_5_ selectivity (**Figure 1A**). [D-Trp^32^]NPY and [Ala^31^, Aib^32^]NPY have been reported to be highly selective and potent agonists of Y_5_ receptor (Parker et al., 2000; Cabrele et al., 2002). However, the most potent and selective activator of the Y_5_ receptor subtype is a chimeric peptide derived from chicken PP, human NPY, and human PP ([cPP^1-7^, NPY^19-23^, His^34^]hPP; Cabrele and Beck-Sickinger, 2000). In addition, Y_5_ receptors display high-affinity to some NPY analogs which also have a considerable affinity for other Y receptor subtypes. [Leu^31^,Pro^34^]pNPY is a Y_1_/Y_4_/Y_5_ selective agonist (Fuhlendorff et al., 1990; Widdowson et al., 1997), whereas the deletion of the first Tyr residue results in the Y_2_/Y_5_ selective agonist NPY (2–36) (Gerald et al., 1996).

Since NPY has been shown to stimulate feeding via the Y_5_ receptor, intensive research has been performed to identify small molecule antagonists of the human Y_5_ receptor as potential feeding suppressors. The first compound that was published as an antagonist of Y_5_ receptor was CGP71683A (**Figure 5B**), which displayed high-affinity and selectivity at the rY_5_ receptor (Criscione et al., 1998). Later studies confirmed the high-affinity and selectivity also for the hY_5_ receptor subtype (Rueeger et al., 2000). Other selective small antagonists at the human Y_5_ receptor include L152,804 (Kanatani et al., 2000a), FMS586 (Kakui et al., 2006), MK-0557 (Erondu et al., 2007), and SCH 500946 (Mullins et al., 2008). In the last 5 years, more than 10 studies have been published presenting newly identified or improved small molecule antagonists of the Y_5_ receptor. This clearly shows the importance of the Y_5_ receptor as an anti-obesity target.

## HOW TO IDENTIFY RELEVANT RESIDUES ON THE RECEPTOR FOR BINDING AND SUBTYPE SELECTIVITY

### GENERAL STRATEGIES

As these receptors consist of 350–450 residues it is impossible to perform a single mutagenesis approach to investigate each amino acid. In order to overcome this problem chimeric receptors can be used, in which fragments of a receptor (e.g., extracellular loops or transmembrane helices) can be exchanged between receptor subtypes. Testing these new constructs with the main agonist from both receptor subtypes can provide information about the importance of one or the other segment, in terms of interaction with the ligand. As soon as an important area in a receptor has been identified, a more detailed study can be carried out using single and multiple mutants, where certain residues of a receptor subtype are exchanged by the ones present on the other receptor subtype of investigation. This strategy allows to find amino acids that may play a role in selectivity to a certain agonist.

When investigating the relevance of the N-terminus, successive truncations or substitutions using tags or spacers can be an elegant method. Furthermore, C-terminal truncations can provide information about segments relevant for internalization. It is known that this receptor part is involved in arrestin-dependent internalization processes of Y_1_, Y_2_, and Y_5_ receptors (Walther et al., 2011). However, single mutagenesis techniques can be used to investigate important residues for the structure or for ligand receptor interactions. Using this approach, certain residues located in extracellular areas of the protein are mutated to Ala or other amino acids in single substitutions. The residues can be chosen according to its location, charge, aromaticity, hydrophobicity. Moreover, 3-D models are also a good tool to select new targets, although mutagenesis data are needed to refine the models and make them more reliable. Once a relevant residue has been identified, double cycle mutagenesis can be used to find the type of interaction that includes both positions. In this technique, peptide analogs containing modifications in positions of interest are investigated with receptor mutants. The aim is to form artificial bonds to proof a native interaction. The introduction of charged residues in the peptide and receptor positions to create a repulsion/attraction, or aromatic residues and hydrophobic residues are feasible ways to prove a ligand receptor interaction. In order to finally prove a ligand-receptor interaction, a reciprocal mutation approach can be followed, where the residue of interest on the peptide side is exchanged by the residue present on the receptor side and *vice versa*. In the case of a critical position or segment, the binding affinity of the native ligand should significantly decrease, whereas in signal transduction assays the EC_50_ or half maximal activation value should increase. Despite all the advantages that these approaches provide, it has to be taken into account that they also present some disadvantages. Thus, when constructing receptor chimeras or receptor mutants, alterations in the receptor structure may arise due to misfolding and therefore may lead to impaired receptor export. Moreover, these modifications might result in a reduced receptor retention time at the cell surface and an enhanced degradation. All together, this might lead to a loss in binding or receptor activity. In order to analyze such phenomena, fluorescence microscopy, cell surface ELISA or radioligand binding studies are a good tool to ensure cell surface expression.

### Y_1_ RECEPTOR

In the past years, much effort has been made to characterize this receptor. Using N-terminal truncations and receptor chimera, it could be elucidated that the N-terminal part of NPY receptors does not participate in the binding pocket. N-terminal truncation in the hY_1_ receptor disrupts the membrane expression; however any eight residues are enough to recover the membrane expression (Lindner et al., 2009).

From all these studies, a number of residues emerged as important for the receptor. First of all, two negatively charged residues are able to establish electrostatic interaction. Asp^2.68^ and Asp^6.59^ were found to be important for the receptor as the peptide loses affinity when mutated to Ala (Sautel et al., 1995, 1996; Kannoa et al., 2001; Sjodin et al., 2006). Furthermore Asp^6.59^ was shown to bind Arg^35^ of pNPY being the first and only proved interaction for this receptor (Merten et al., 2007). Other residues that appeared to be important in several studies are Tyr^2.64^, Phe^6.58^, and His^7.31^ (Sautel et al., 1995, 1996; Kannoa et al., 2001; Sjodin et al., 2006), although a direct interaction was never established for any of the amino acids of the ligand. It was suggested that these residues form a hydrophobic pocket in the receptor. Further investigations using the Y_1_ receptor antagonist BIBP 3226 showed that Tyr^2.64^ and His^7.31^ did not affect the conformation of the receptor in a major way (Sautel et al., 1996) as the antagonist was perfectly bound. Taken together, it is very likely that position 6.58 and 7.31 interact with a C-terminally located amino acid, on the other hand it is unlikely that Tyr^2.64^ interacts with the C-terminus as it seems to be too far from the other two residues.

**FIGURE 6:**
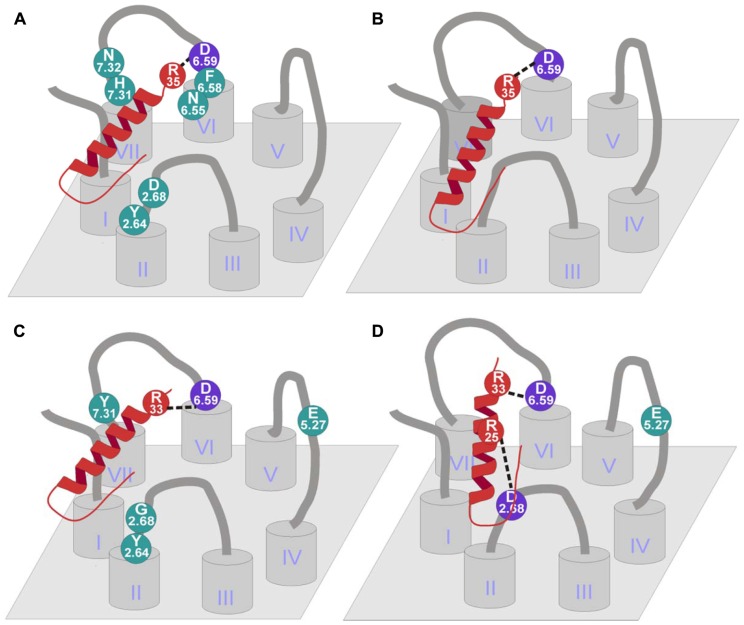
**Binding mode of NPY receptors**. **(A)** hY_1_ receptor binding mode (Sautel et al., 1995, 1996; Kannoa et al., 2001; Sjodin et al., 2006; Merten et al., 2007); **(B)** hY_4_ receptor binding mode (Merten et al., 2007); **(C)** hY_2_ receptor binding mode (Merten et al., 2007; Akerberg et al., 2010); **(D)** hY_5_ receptor binding mode (Merten et al., 2007; Lindner et al., 2008b).

Other relevant residues of the receptor are Trp^6.60^, Asn^6.55^, and Asn^7.32^ (Kannoa et al., 2001). Although position 6.55 is in a slightly deeper position, Asn^6.55^ and Asn^7.32^ showed a loss in PYY binding and also in antagonist binding suggesting that they could play a role in ligand binding (**Figure 6A**).

Studies with antagonists indicate that the binding of these compounds differs depending on the ligand between transmembrane helices 3 and 7. Taking all the data into consideration, it can be assumed that the binding pattern of the native ligands and the small antagonists overlaps in TM6 because several residues have been found to be relevant in both cases.

### Y_2_ RECEPTOR

In this receptor subtype, the N-terminus does not play a role in membrane expression and it does not participate in a subtype specific binding pocket. However, it does play a role in agonist induced internalization processes since the complete truncation slowed down the process, although it could be seen that the exchange of the N-terminal fragment by the hY_1_ receptor or hY_5_ receptor fragment did not affect ligand dependent internalization (Lindner et al., 2009).

Mutagenesis studies to identify residues that contribute to ligand binding in the Y_2_ receptor were initially motivated by the finding that human and chicken Y_2_ receptors show a significantly different pharmacological profile. The chicken Y_2_ receptor is able to bind [Leu^31^,Pro^34^]-NPY, a peptide agonist selective for mammalian Y_1_/Y_4_/Y_5_ receptors, but was unable to bind BIIE0246, a small molecule antagonist for mammalian Y_2_ receptors (Salaneck et al., 2000). Sequence comparison and reciprocal mutagenesis revealed three residues in transmembrane helices 3, 5, and 6 that contribute to the binding of BIIE0246. Individual and combined substitution of Gln^3.37^, Leu^5.51^, and Leu^6.51^ in the hY_2_ receptor decreased the affinity for BIIE0246 to a chY_2_-like level, whereas substitution of the corresponding residues in the chY_2_ by the human residues increased the affinity for BIIE0246 (Berglund et al., 2002). Further mutagenesis studies on the human Y_2_ receptor revealed interaction partners for the native peptidic ligand NPY. Several acidic residues have been tested for their importance for NPY binding. Glu^5.27^ and Asp^6.59^ turned out to be highly important for the binding of NPY (**Figure 6C**). While Asp^6.59^ is important for all Y receptor subtypes, Glu^5.27^ only plays a role in the Y_2_ receptor. Both receptor mutants were tested in a signal transduction assay using pNPY, [Ala^25^]pNPY, [Ala^33^]pNPY, and [Ala^35^]pNPY to identify the interaction partner of the two acidic residues in the peptide. It could be shown that Asp^6.59^ interacts with Arg^33^ of the peptidic ligand in the Y_2_ and Y_5_ receptors, whereas the interaction partner in Y_1_ and Y_4_ receptors is Arg^35^. However, no direct interaction partner could be identified for Glu^5.27^ (Merten et al., 2007). More recent studies investigated additional residues in the Y_2_ receptor for their impact on the binding of pPYY, pNPY, hPYY (3–36), pNPY(13–36), and the non-peptidic antagonist BIIE0246 (Akerberg et al., 2010; Fallmar et al., 2011). The residues tested, namely Tyr^2.64^, Gly^2.68^, Thr^3.40^, Leu^4.60^, Gln^6.55^, Val^6.58^, and Tyr^7.31^, were chosen by similarity to residues in the Y_1_ receptor subtype, which were proven to be important for ligand binding in this receptor subtype. It could be shown, that of the tested residues, only Tyr^2.64^ participates in the binding of all tested peptidic ligands and the non-peptidic antagonist BIIE0246. The substitution of this residue to Ala resulted in a five- to ninefold reduction in affinity (Akerberg et al., 2010).

The individual substitution of Tyr^7.31^ by Ala and Gly^2.68^ by the bigger and more polar residue Asn revealed a lower affinity only for the truncated peptide agonists. The authors hypothesize that Tyr^7.31^ does not play a role in binding of the full-length peptide, but may contribute to a compensatory interaction for ligands that lack the N-terminal residues. Furthermore, the authors could show that an introduction of a His residue in position 7.31 (the corresponding residue in Y_1_ receptor) completely abolished the binding of [^125^I]-pPYY (Akerberg et al., 2010). These findings are somewhat unexpected, since this His residue was shown to be involved in ligand binding in the Y_1_ receptor (Sjodin et al., 2006). This indicates that position 7.31 is important in both receptor subtypes, but may have different modes of action (Akerberg et al., 2010). For position 2.68, a mode of binding is proposed in which the lack of Asp (a residue common to all other Y receptor subtypes at this position) contributes to the selectivity of truncated peptides [e.g., NPY(3–36)] for the Y_2_ receptor (Fallmar et al., 2011). The Leu^4.60^Ala mutant showed a slightly decreased affinity for hPYY(3–36) and a strong loss of affinity for BIIE0246, which may be caused by a weakened or lost hydrophobic interaction. This indicates that this residue is highly important for antagonist binding. The corresponding position in Y_1_ receptor (Phe^4.60^) has been shown to be involved in the binding of [^125^I]NPY, [^3^H]BIBP3226 (Sautel et al., 1996) as well as [^3^H]J-104870 (Kannoa et al., 2001). This indicates that position 4.60 is involved in the binding of small molecule antagonists at both receptor subtypes, Y_1_ and Y_2_.

The Y_2_ receptor mutants Thr^3.40^Ile and Gln^6.55^Ala showed increased affinity for pNPY and hPYY(3–36), but decreased affinity for the non-peptidic antagonist BIIE0246. Taken into account that these positions are located deeper in the transmembrane part of the receptor, an indirect effect on the binding of peptidic ligands would be the most likely explanation. However, the decreased binding of BIIE0246 may be also explained by a different binding pocket for small molecule antagonists, located more deeply in the transmembrane (Fallmar et al., 2011) and surrounded not only by Thr^3.40^ and Gln^6.55^, but also by the nearby residue Gln^3.37^, which was earlier shown to participate in the binding of BIIE0246 earlier (Berglund et al., 2002).

### Y_4_ RECEPTOR

N-terminal truncations and substitutions revealed the importance of this fragment for membrane expression and indicated that the N-terminus is not involved in forming a specific binding pocket. It is likely that this part could stabilize the TM1 to ensure the correct receptor structure (Lindner et al., 2009).

Two positions were investigated to find the binding partners on the ligand side (Merten et al., 2007). Glu^5.24^ was mutated to Ala in order to test the influence of the side chain, Glu^5.24^Ala showed a threefold loss in potency. On the other hand, Asp^6.59^ was mutated to Ala, Glu, Asn, and Arg to test the influence of charge and length of the side chain. The mutation to Ala showed a complete loss of both binding and activity, the exchange to Glu displayed wild-type-like binding and activation. In addition, the mutation to Asn showed indeterminate binding and a 200-fold loss in activation. Finally the exchange to Arg resulted in a dramatic loss in potency (>600-fold) and in no detectable binding (**Figure 6B**).

### Y_5_ RECEPTOR

The Y_5_ subtype N-terminus could play a role in ligand binding, since the partial truncation of the segment produced a loss in activation. Interestingly, the receptor remains on the membrane even when the complete N-terminus is removed (Lindner et al., 2009).

Only few mutagenesis studies have been published so far for the human Y_5_ receptor. Merten et al. (2007) exchanged three acidic residues in the extracellular domains of Y_5_ receptor. While the Asp^6.62^Ala mutant showed wild-type-like pharmacological properties, Asp^6.59^Ala and Glu^5.27^Ala displayed a dramatically reduced affinity for NPY. Additional residues were investigated by Lindner et al. (2008b), resulting in identification of a third acidic residue (Asp^2.68^) which is important for ligand binding at the Y_5_ receptor. These Ala-mutants have also been tested with NPY analogs in which theTyr^27^, Tyr^36^, and the Arg residues at position 25, 33, and 35 were individually substituted by Ala (**Figure 6D**). This approach revealed no further loss of affinity for [Ala^33^]pNPY on the Asp^6.59^Ala mutant of the receptor, indicating a direct interaction between Ala^33^ of the peptide and Asp^6.59^ of the receptor. Similarly, Arg^25^ of the NPY peptide could be identified as the interaction partner for Asp^2.68^ of the receptor (Lindner et al., 2008b).

## CONCLUSION AND PERSPECTIVES

The NPY system has been extensively characterized in the last years. The modulation of actions mediated by the distinct receptors like, e.g., its involvement in obesity, cancer, and epilepsy are of great importance. Therefore, the development of receptor subtype-selective ligands and structure-activity relationship studies have been a major objective in the past years. Primarily, amino acid scans and truncations have identified the important residues and areas of the ligand with respect to binding at each receptor. The Y receptors have been extensively studied, several important residues have been characterized and some of the binding pockets have been partially characterized. Two subtype-selective interactions have been elucidated so far. A similar binding mode has been identified on NPY receptors, where a common residue Asp^6.59^ binds to one of the two C-terminally located Arg of the peptide depending on the receptor subtype. Moreover, a second binding interaction has been found on the Y_5_ receptor where Asp^2.68^ located at the top of TM2 interacts with Arg^25^ of the peptide (Merten et al., 2007; Lindner et al., 2008b). This finding would suggest that probably a second interaction could take place in other receptor subtypes. Nevertheless, further investigations have to be performed. It is likely that more interactions between the receptors and the peptides could occur, therefore structure activity relationship studies are still a focus of interest.

The design of short analogs and antagonists have confirmed these findings, indicating that this is a great tool to modulate and study the receptors. Some promising progresses have been achieved in cancer diagnosis using Y_1_ receptor selective short ligands. However the development of short analogs for treatment of this pathology still remains challenging. Also in anti-obesity drugs, Y_2_/Y_4_receptor selective agonists are in progress and currently in clinical trials of Phase I/II. On the basis of well studied characteristics accounting for receptor subtype selectivity, it is likely that subsequent investigations could be focused on the improvement of pharmacological properties such as stability and half-life. In addition the development of more potent selective ligands might be a focus of interest.

## Conflict of Interest Statement

The authors declare that the research was conducted in the absence of any commercial or financial relationships that could be construed as a potential conflict of interest.
